# Scientific highlights of the 9th ESWI Influenza Conference

**DOI:** 10.1186/s42522-024-00099-4

**Published:** 2024-04-02

**Authors:** Leslie Reperant, Colin A. Russell, Albert Osterhaus

**Affiliations:** 1 Pikado BV, Utrechtse Heuvelrug, the Netherlands; 2 Department of Medical Microbiology and Infection Prevention, Amsterdam University Medical Center, Amsterdam, The Netherlands; 3 Center of Infection Medicine and Zoonosis Research and the University of Veterinary Medicine Hannover, Hannover, Germany

## Abstract

The European Scientific Working Group on Influenza (ESWI) held the 9th ESWI Influenza Conference in Valencia from 17—20 September 2023. Here we provide a summary of twelve key presentations, covering major topics on influenza virus, respiratory syncytial virus (RSV) and SARS coronavirus 2 (SARS-CoV-2) including: infection processes beyond acute respiratory disease, long COVID, vaccines against influenza and RSV, the implications of the potential extinction of influenza B virus Yamagata lineage, and the threats posed by zoonotic highly pathogenic avian influenza viruses.

## Introduction

The European Scientific Working Group on Influenza (ESWI) held the 9th ESWI Influenza Conference in Valencia from 17—20 September 2023. It gathered over 1,000 attendees from 72 countries and gave the floor to renowned and early career scientists, public health experts and healthcare professionals. With promising developments in the field of respiratory viral infections, the conference sessions covered hot topics on seasonal, pandemic, and zoonotic influenza viruses, as well as other respiratory viruses such as respiratory syncytial virus (RSV) and SARS coronavirus 2 (SARS-CoV-2). As Colin Russell, the new chair of ESWI, indicated, ESWI as an organization is “no longer a society that’s focused on flu and standardising laboratory techniques. Over the last 20 years, we’ve had two pandemics, [pandemic influenza in 2009 and COVID-19 in 2020] the consequences of which last with us today. We have also seen the increase in potential for intervening with and treating RSV. The ESWI conference has grown into a keystone event discussing the latest advances in these major respiratory viral infections.”

## Beyond acute respiratory viral infections: long-term effects and secondary bacterial infections

Peter Openshaw reminisced in the conference opening lecture (Immune Responses to Respiratory Viruses and ‘Long-Haul’ Disease) that even when first appointed as a consultant physician at a London teaching hospital, he suspected that many of the peculiar cases of diseases of unknown etiology, whether fibrotic or inflammatory, appeared suspiciously related to infection. These might be common infections triggering rare events, or rare but occult infections that presented as inflammatory processes. Already fascinated by immune responses and inflammatory responses to viruses, Openshaw grew intrigued by the possible delayed effects of infection.

Besides influenza viruses, respiratory viruses with recognized long-term effects include RSV and SARS-CoV-2. RSV Bronchiolitis is essentially an inflammatory condition, RSV is known to manipulate the host immune response via many of its proteins [13, 29]. Children who have recovered from bronchiolitis often have recurrent wheeze with asthma diagnosis [39], and specifically blocking RSV infection by administering palivizumab may result in a significant reduction in the frequency of wheeze in the first year of life [4, 21]. Openshaw concluded that the advances of long-acting antibodies and vaccines to prevent RSV disease in childhood hold the potential to promote long-term respiratory health by interfering with these effects.

Openshaw also presented the results of the large collaborative Post-Hospitalisation COVID-19 study (PHOSP-COVID) in the UK that allowed to categorize Long COVID into different symptom complexes, including gastrointestinal, fatigue, respiratory, cardiac, and neuropsychiatric symptoms [7, 18]. The study involved detailed inflammatory profiling to uncover potential mechanisms driving Long COVID and associated combinations of symptoms. Penalized logistic regression was applied to identify trends in the data, such as the association of myeloid inflammation and complement activation with certain symptoms. Rather than identifying markers of disease as potential diagnostic tools, Openshaw emphasized that this study pointed to the importance of considering individualized treatments for different types of Long COVID rather than a one-size-fits-all approach.

Worse outcomes of COVID-19 are often associated with secondary bacterial infection. In her presentation (Host Transcriptomics and Machine Learning provide a Novel Forward Predictive Model for Bacterial Infections in Patients with COVID-19), Kirsty Short discussed a model to predict the development of secondary bacterial infections more than 24 h after hospital admission (Carney et al., under review). The approach included differential gene expression analysis on samples collected early during the pandemic. Machine learning (specifically LASSO) was employed to select predictive genes from a minimal set. Seven genes associated with tissue damage and inflammation were identified, resulting in a model of gene expression and patient data with high predictive value, awaiting validation in a contemporary, vaccinated cohort. Short concluded that such models could help reduce the use of antibiotics in viral infections.

## The importance of T cell immunity

Katherine Kedzierska delivered a keynote address (Human Immunity in Respiratory Virus Infection and Vaccination) highlighting the research carried out at her lab at the Peter Doherty Institute for Infection and Immunity, on understanding immunity to seasonal, pandemic, and emerging viruses, immune perturbations in high-risk groups, recovery mechanisms, and comparing natural to vaccine-induced immunity. A physical cohort established in 2014 and capturing data from patients on hospital admission enabled the analysis of immune responses in COVID-19 patients, early during the pandemic [17, 41]. They observed regulated cytokine responses and broad, robust but transient immune responses across both innate and adaptive immune responses in mild to moderate cases, but dysregulated responses linked with hyperactivation of both innate and adaptive responses in severe cases, suggesting a detrimental effect on the immune system. Interestingly, in healthy pregnant women a substantial activation of the first line of defense was measured that remained tightly regulated and did not significantly increase after SARS-CoV-2 infection [11]. This differential regulation of innate immunity in pregnant vs non-pregnant women may, at least in part, explain increased susceptibility of pregnant women to respiratory viruses.

Focusing on T cell responses, specifically killer CD8 + T cells, which are known to be essential in controlling infection, Kedzierska’s team identified new SARS-CoV-2 epitopes, including immunodominant epitopes associated with lower viral replication and milder disease [12, 22, 28, 31, 35, 37], highlighting the importance of CD8 + T cells in preventing severe COVID-19. Longitudinal studies revealed the persistence of these cells for up to two years.

Transitioning to COVID-19 vaccination research, Kedzierska discussed efforts to define immune responses in high-risk patients and First Nation people [27, 49, 50]. Indigenous populations globally are disproportionately affected by infectious diseases. As the vaccine rollout started in March 2021, Aboriginal people were identified as a priority group. But without vaccine information in Aboriginal language, fear about COVID-19 and the vaccine was spreading faster than the virus in those communities. This prompted the deployment of a community engagement study that generated and produced videos in ten Aboriginal languages to inform these communities about COVID-19 vaccination. This critical engagement effort led to great enthusiasm for COVID vaccination in Aboriginal communities and in participating in research studies on immune responses.

Kedzierska’s team found significantly lower antibody levels in Indigenous people at day 28 after the second vaccination dose, compared to non-Indigenous people. They examined glycosylation patterns of antibodies, revealing lower galactosylation and lower overall antibody responses in Indigenous individuals with comorbidities, such as diabetes and renal disease [49, 50]. However, similar results were seen in non-Indigenous people with similar comorbidities, pointing to comorbidities rather than ethnicity as the drivers of lower immune responses. This work, however, has important implications for First Nations people globally as Indigenous populations have disproportionately higher rates of chronic diseases worldwide.

Kedzierska concluded by emphasizing the importance of understanding immune responses in Indigenous populations, addressing the impact of chronic diseases, and showcasing an artwork by Zoe Fitzpatrick reflecting the collaborative efforts of researchers, health professionals, and Indigenous communities in finding better ways to protect chronically unwell people.

Carolien van de Sandt, who works in Kedzierska’s lab, was awarded with the Claude Hannoun Prize for Best Body of Work. Her presentation (Dynamics of CD8 + T cell immunity to Circulating and Pandemic Viruses) further highlighted the protective role of CD8 + T cells against severe respiratory disease, this time caused by influenza virus infection [44]. She highlighted the role of CD8 T cells in recognizing conserved epitopes and providing protection against zoonotic influenza viruses, by enhancing viral clearance and reducing disease severity [47]. Importantly, these T cell populations were shown to be long-lived and of potential protective value in the event of an influenza pandemic [43].

Van de Sandt discussed the impact of HLA alleles on CD8 T cell responses, specifically HLA-A*68, which is present in 5–25% of the human population, especially in indigenous people, and associated with more severe respiratory disease upon infection [42]. HLA-A*68 presents an unusually long influenza virus peptide that is highly conserved across subtypes. However, such a bulky epitope proved quite unstable and difficult to crystalize, revealing how recognizing this cognate epitope proved equally challenging for T cell receptors, resulting in low T cell immune responses.

Van de Sandt further explored how changes in CD8 + T cell frequency, phenotype, and clonal composition unravel with age, demonstrating differences in CD8 T cell responses in children, adults, and older adults [45]. Surprisingly, influenza virus A and B specific T cells did not present signs of immunosenescence, exhaustion, or terminal differentiation in older adults. The elderly gene expression profiles were more similar to those of newborns and children and did not have a terminally differentiated state. TCR of older adults had a higher activation threshold, lower binding affinity, and limited ability to recognize peptide variants. Van de Sandt’s work during the COVID-19 pandemic revealed similar age-associated differences in the immune response against SARS-CoV-2. Her research delved into the antibody and CD8 T cell responses in children and adults, shedding light on the differences in immune profiles [38]. Children had a more polyreactive antibody response compared to older adults, especially in terms of their phagocytosis abilities. Children also benefited from a very strong innate immune response that helped eradicate the infection much faster than in adults [25]. However, the magnitude and clonal expansion of the T cell response were larger in adults [36]. Van de Sandt further discussed the impact of immunomodulatory treatments on CD8 + T cell responses in COVID-19 patients, demonstrating that they did not prevent immunosuppressed patients from mounting robust T cell responses upon vaccination.

## Vaccine advances against influenza and RSV

Jenna Guthmiller was the recipient of the Young Scientist Vaccine Innovation Award and addressed the challenges in generating broadly protective influenza vaccines, emphasizing the impact of immunodominance and original antigenic sin (The Co-Evolution of Influenza Viruses and the Antibodies that neutralize them).

Her presentation highlighted efforts to identify antibodies from memory B cells against broadly protective epitopes, particularly in response to H1N1 viruses. She discussed the impact of pre-existing immunity in shaping B cell specificities induced by infection or vaccination. Guthmiller used monoclonal antibodies to interrogate their epitope specificity, breadth, and function, and showed that individuals with low pre-existing antibodies against influenza virus variable epitopes were more likely to generate antibody responses against conserved epitopes [9, 10]. Armed with this knowledge, she explored a vaccine platform designed to reduce pre-existing antibodies against variable regions (chimeric HA vaccination), showing promising results in inducing antibodies against the conserved stock domain [9, 24].

Guthmiller addressed the complexity of influenza virus evolution, examining evidence of antigenic drift in broadly protective epitopes. She presented findings related to lateral patch-targeting antibodies, noting an age-dependent aspect to their efficacy and discussing mutations that impacted their neutralizing capabilities. She showed that the monoclonal antibodies targeting the lateral patch can drive the antigenic drift in vitro*,* as also seen in nature. She concluded with insights into clonal expansion and the potential for B cells to redeem themselves through affinity maturation, essentially losing affinity for whatever is antigenically drifting, providing hope for the continued viability of HA antibody targets for influenza vaccines.

Marta Picciolato provided an overview of the efficacy of a maternal RSV vaccine by showing the efficacy of an unadjuvanted maternal RSV prefusion F protein-based vaccine in infants in an interim analysis up to six months post-birth. The study aimed at evaluating the efficacy of a single dose of an RSV prefusion F-based vaccine in pregnant women in preventing RSV lower respiratory tract disease in infants up to six months of age, through the boosting of maternal antibodies. The trial, named RSV MAT-009 (NCT04605159), a phase 3, double-blind, 2:1 randomized, placebo-controlled, multi-country trial, started in November 2020 and enrollment and vaccination were stopped in February 2022 following the observation of an imbalance in preterm births and neonatal deaths between the vaccine and placebo groups. Enrollment and vaccination were halted, and the study was unblinded. The safety signal related to neonatal deaths was found not to be an independent signal but be associated with the preterm birth signal.

The baseline characteristics of participants, including age, gestational age at vaccination, race, and delivery interval, were well-balanced. The birth characteristics of infants were also balanced between the vaccine and placebo groups. Active and passive surveillance methods and specific assessment visits with nasal swabbing were employed to assess RSV lower respiratory tract disease in infants. The primary efficacy analysis revealed a vaccine efficacy of 65.5% against RSV lower respiratory tract disease and 69% against severe RSV lower respiratory tract disease. Further analysis showed similar efficacy against RSV strains A and B.

Picciolato concluded that a single dose of the RSV maternal vaccine administered to maternal participants during the late second or third trimester of pregnancy provided protection against medically assessed RSV lower respiratory tract diseases in their infants up to six months of age. However, the results can only be interpreted as descriptive due to the unblinding of the study following the enrollment and vaccination stop and may be associated with potential performance or detection biases.

## The extinction of influenza B virus Yamagata lineage: a panel discussion with Ben Cowling and Wenqing Zhang

Moderated by Ab Osterhaus, this panel addressed the consequences of the apparent decline or extinction of B/Yamagata lineage in seasonal influenza viruses. Wenqing Zhang provided an update on the surveillance data, indicating a dramatic reduction in the proportion of B/Yamagata lineage among seasonal strains since 2021. The discussion revolved around the potential exclusion of B/Yamagata from influenza vaccines, as considered during this year’s WHO vaccine composition consultation meetings. The decision to exclude B/Yamagata is seen as a matter of when rather than if, and the participants expressed the need for a well-organized approach involving public and private sectors, regulatory agencies, vaccine manufacturers, and academia to address this issue.

Ben Cowling further emphasized the need for defining the criteria and timeframe for declaring the virus extinct, potential risks of resurgence, risk mitigation considerations, especially for live attenuated influenza vaccines, and the time required for regulatory procedures if a switch from quadrivalent to trivalent vaccines is considered. The latter may require a process to reactivate or reapply for trivalent influenza vaccine registrations in many countries. The timelines need to be carefully considered. Wenqing Zhang pointed out that confirming lineage extinction and deciding on vaccine composition are two separate issues, and regulatory agencies and manufacturers are discussing risk mitigation processes in the case of an outbreak in the future, including the possible use of a monovalent vaccine. Regulators and manufacturers are also considering practical solutions for the risk associated with reassortment of live attenuated vaccines. Regarding the timelines, and since regulatory procedures may take anywhere between two and 48 months, Wenqing Zhang agreed that preparation of all stakeholders in the field will be key.

## Nonhuman primate and human challenge models

To further advance preclinical testing of novel influenza vaccines, Maya Sangesland (Convergent Recognition of Antigenic Supersites Within Influenza Hemagglutinin Stem in Nonhuman primates) presented her work on understanding B cell and antibody responses to influenza hemagglutinin (HA) stem in non-human primates (NHPs) and addressed whether NHPs can mirror human immune responses to vaccines. In humans, antibody responses to the stem epitope of HA predominantly use the VH1-69 gene, and are broadly protective [19, 30, 40, 48]. While humans have established pathways for eliciting antibodies targeting this region, NHPs lack certain critical contact residues and were previously thought unable to recapitulate this protective response.

In a clinical trial (NCT03814720) using a nanoparticle vaccine displaying stabilized H1 stem, it was recently shown that humans generate two distinct B cell responses: one targeting the central stem and another targeting the anchor epitope [2]. Surprisingly, Sangesland found that vaccinated NHPs generated B cell populations are similar to those seen in humans, despite differences in critical contact residues. One population, analogous to the central stem-targeting response in humans, predominantly used VH1-138, a homologue to human VH1-69. The other population showed a bias towards VH3, associated with anchor-targeting antibody responses in humans. Further epitope specificity analysis suggested that NHPs displayed convergence in VH gene repertoires and epitope specificities within the two B cell populations. Functional assessment of the antibodies demonstrated broadly neutralizing activity of the central stem-targeting antibodies against different influenza viruses. Anchor-targeting antibodies on the other hand, were less broad and functional, in contrast to human anchor-targeting antibodies. Structural analysis suggested that NHP antibodies targeted sites similar to those targeted in humans.

Sangesland also explored the protective efficacy of isolated monoclonal antibodies through prophylactic challenge experiments in mice. Both central stem- and anchor-targeting antibodies, despite the latter being non-neutralizing, demonstrated protective efficacy. In conclusion, the findings strongly supported the ability of NHPs to mirror certain canonical human immune responses to influenza, supporting their use as a model for preclinical testing of influenza vaccines and enhancing the understanding of influenza infection immunology.

In a dedicated session on Controlled Human Infection Models (CHIM), Christopher Chiu and Adrian Wildfire discussed their views on the strengths and weaknesses of such models for respiratory virus infections. Chiu introduced the topic and provided a brief history of CHIMs, emphasizing their role in understanding viral effects, strain, dose, and delivery [34]. He focused his presentation on how CHIMs can help identifying novel correlates of protection for respiratory viruses, particularly highlighting the role of T cell immunity.

The strengths of CHIMs were discussed, including the use of well-defined viral strains, controlled settings, and the ability to achieve consistent infection rates. Chiu emphasized the importance of longitudinal studies and the ability to assess pre-infection and early post-exposure periods for understanding immunity. He highlighted the unique ability of CHIMs to allow sampling usually inaccessible anatomical compartments, such as the upper and lower respiratory tracts, providing insights into tissue-resident T cells.

Chiu presented data from CHIM studies on RSV and influenza virus [14, 26], showcasing the enrichment of tissue-resident T cells in the lower respiratory tract and their correlation with reduced severity of disease and reduced viral load. Omics technologies were essential to analyze differentially expressed gene pathways in tissue-resident T cells, demonstrating the production of chemokines and cytokines typically associated with innate immunity.

Chiu led the development of a SARS-CoV-2 human challenge model during the pandemic, aiming to demonstrate the kinetics of viral load and symptomatology, highlighting the granularity of data for mathematical modeling [3, 15, 32]. T cell activation negatively correlated with viral load and duration of viral shedding. Most innovatively, the model allowed the study of immune responses in the early pre- and post-exposure period, with single-cell RNA-seq data revealing rapid T cell influx in exposed but uninfected individuals, which was not seen in individuals who went on to develop sustained infection.

Wildfire introduced his talk with a meta-analysis that provided comprehensive evidence of the decrease in hospitalizations and case fatality ratios (CFR) among individuals vaccinated against SARS-CoV-2 [52]. He highlighted the critical role of cytokines and chemokines in disease severity and that lower levels of these immune mediators in vaccinated individuals offered a relevant mechanism contributing to preventing severe disease. However, despite CFR falling, transmission of SARS-CoV-2 persisted. Breaking transmission through vaccination will be essential to thwart a pandemic, keeping robust indices of disease severity suppressed will not be sufficient.

In the presence of novel SARS-CoV-2 variants, the data of another contact-tracing study suggested largely similar attack rates between vaccinated and unvaccinated individuals [16]. However, vaccinated individuals cleared the virus more quickly than the unvaccinated. Surprisingly though, they still had a similar peak viral load to unvaccinated individuals, indicating continued shedding. This may be due to transient mucosal immunity induced by intramuscular vaccination.

To introduce novel vaccines that can effectively break transmission, using secondary attack rates in addition to disease incidence will be necessary. Collected evidence supports the conception that the dose of the exposure to SARS-CoV-2, as the product of both the intensity and the duration of exposure, has a positive connection with the viral load as well as the frequency and the severity of the resulting disease. However, a SARS-CoV-2 CHIM study in 2021 identified participants with relatively low nasal viral load who emitted large amounts of virus. This finding suggested that measuring viral emissions is a better surrogate for infectiousness [51].

To prove that new vaccines are better at breaking transmission, even a high secondary attack rate of 30% may not be sufficient to allow for field trials to be affordable or possible. Furthermore, markers relevant to estimating vaccine efficacy against transmission are not known nor mentioned by regulatory agencies. CHIM transmission studies may be one of the few methods of estimating efficacy against viral transmission with a reasonable cost and timeline.

## Zoonotic threats

Lastly, Ron Fouchier (HPAI H5 Viruses in Wild Migratory Birds in the Netherlands) and Reina Sikkema (HPAI H5 Virus Infections and Antibodies in Wild Carnivores in the Netherlands, 2020–2022) reviewed the threats posed by zoonotic highly pathogenic avian influenza (HPAI) viruses of the H5 subtype currently causing a pandemic in wild and domestic birds. Fouchier provided a historical perspective, noting the virus's origin in poultry from mainland China and its subsequent global spread through trade, migratory birds, and poultry, affecting various species, including humans.

Fouchier reviewed the current global situation, particularly in Europe, with an emphasis on wild birds. The spread of the virus into Europe is detailed, with a focus on different virus lineages and their routes of entry [46]. He highlighted the role of long-distance migratory ducks, such as Eurasian wigeon, as potential vectors for introducing the virus. Changes in the pattern of outbreaks were noted, with the virus becoming more enzootic and causing continuous spread in bird populations.

Fouchier also covered unexpected events, such as the spread of H5N1 viruses to North America, affecting seabirds and causing significant mortality [5, 8]. The emergence of new viral lineages, like the BB virus, was discussed, along with its apparent adaptation to gull and tern species [33].

Fouchier emphasized the need for active and passive surveillance of wild and domestic animals, including mammals, to understand and mitigate the impact of HPAI H5N1 virus. He concluded by addressing the current threat to vulnerable wildlife species, the ongoing circulation of the virus in wild birds, the relevance of viral genotypes for epidemiology, and the occurrence of severe neurological disease in both birds and mammals, prompting a call for further investigation. The risk to humans is acknowledged, but the focus remains on understanding and managing the virus's impact on wild animal populations.

Reina Sikkema focused on the transmission of HPAI H5 viruses to wild carnivores, including terrestrial species like foxes, badgers, and stone martens. She highlighted the importance of monitoring influenza in various species due to potential mammal-to-mammal transmission. Large outbreaks have been observed in mink fur farms, where close proximity enhances the risk of transmission. Her study aimed at investigating true exposure rates and incidence in wild carnivores and the disease caused by circulating viruses in wild carnivores.

The research, spanning from 2020 to 2022, involved citizen science reporting and pathological examinations of reported dead or ill wild carnivores [6]. A total of 20 out of 563 animals tested positive for H5 virus RNA, with foxes (*Vulpes vulpes*), polecats (*Mustela putorius),* and stone martens (*Martes foina*) being the most frequently infected species. Four animals showed neurological signs. An increasing incidence was noted throughout the study period, and molecular analysis revealed specific mutations marking possible adaptation in some virus strains. Serological studies using blood clots from lung cavities furthermore suggested a high, and increasing, exposure rate, with 20% of the animals showing specific antibodies for H5 influenza, pointing to possible asymptomatic infections, the majority of which without neurological complications. High exposure rates were also evidenced in historic wild carnivore serum samples (2016–2017) indicating that infections may have been missed in the past.

Sikkema emphasized the need for increased surveillance, especially in species kept in close quarters like mink farms, as they pose significant risks for both avian influenza and SARS-CoV-2 transmission, mammalian adaptation, and spillover to humans [1, 20, 23].

## Conclusion

The 9th ESWI Influenza Conference covered all major topics in acute respiratory viral infections, providing a successful venue for disseminating the field’s state of the art. The 10th ESWI Influenza Conference will be held from 19 to 22 October 2025 in Valencia, Spain.
